# Tomato Chlorotic Spot Virus (TCSV) Putatively Incorporated a Genomic Segment of Groundnut Ringspot Virus (GRSV) Upon a Reassortment Event

**DOI:** 10.3390/v11020187

**Published:** 2019-02-22

**Authors:** João Marcos Fagundes Silva, Athos Silva de Oliveira, Mariana Martins Severo de Almeida, Richard Kormelink, Tatsuya Nagata, Renato Oliveira Resende

**Affiliations:** 1 Department of Cell Biology, Institute of Biological Sciences, University of Brasília (UnB), Brasília 70910-900, Brazil; joaomarcos.fagundes@gmail.com (J.M.F.S.); athos.so@gmail.com (A.S.d.O.); mariana_martins12@yahoo.com.br (M.M.S.d.A.); tatsuya@unb.br (T.N.); 2 Laboratory of Virology, Wageningen University (WUR), Wageningen 6708 PB, The Netherlands; richard.kormelink@wur.nl

**Keywords:** tospovirus, tomato chlorotic spot virus, groundnut ringspot virus, virus evolution, reassortment

## Abstract

Tomato chlorotic spot virus (TCSV) and groundnut ringspot virus (GRSV) share several genetic and biological traits. Both of them belong to the genus *Tospovirus* (family *Peribunyaviridae*), which is composed by viruses with tripartite RNA genome that infect plants and are transmitted by thrips (order Thysanoptera). Previous studies have suggested several reassortment events between these two viruses, and some speculated that they may share one of their genomic segments. To better understand the intimate evolutionary history of these two viruses, we sequenced the genomes of the first TCSV and GRSV isolates ever reported. Our analyses show that TCSV and GRSV isolates indeed share one of their genomic segments, suggesting that one of those viruses may have emerged upon a reassortment event. Based on a series of phylogenetic and nucleotide diversity analyses, we conclude that the parental genotype of the M segment of TCSV was either eliminated due to a reassortment with GRSV or it still remains to be identified.

## 1. Introduction

The tospoviruses have a great impact on agriculture since they can cause from mild to severe symptoms in their plant hosts [1]. These viruses have recently been reclassified within the *Tospovirus* (family *Peribunyaviridae*; order *Bunyavirales*), a genus that solely encompasses the “bunyaviruses” that have plants as hosts and are propagatively transmitted by thrips (order Thysanoptera) [2,3]. Since they have a tripartite single-stranded RNA genome, each segment is named according to its size as small (S), medium (M), or large (L) RNAs [4,5]. While the L RNA is of negative polarity, both the S and M segments contain an ambisense gene arrangement.

The main criterion for determination of new tospovirus species resides in the amino acid (aa) sequence of the nucleocapsid (N) protein that is encoded in the S RNA [5]. New isolates can only be recognized as belonging to a new species when their N protein shares less than 90% amino acid (aa) sequence identity with members of established species. Besides the N protein, the S RNA codes for a nonstructural protein (NSs) with RNA silencing suppression activity [6]. The M RNA codes for a cell-to-cell movement protein (NSm) and the precursor (GP) to the glycoproteins (Gn and Gc), while the L RNA codes for an RNA-dependent RNA polymerase (RdRp) [7,8]. Although the N protein demarcates new species, phylogenetic trees based on any of the abovementioned proteins usually tell the evolutionary history of tospoviruses since the taxa tend to cluster similarly [9]. In case this is not observed, a reassortment event may likely be the cause for this incongruence. 

Tomato chlorotic spot virus (TCSV) and groundnut ringspot virus (GRSV), although serologically related, were first suggested as members of different tospovirus species in the early 1990s after sequencing the *N* genes of isolates BR-03 (TCSV) and SA-05 (GRSV). Both isolates were initially identified from infected tomato in Brazil and groundnut in South Africa, respectively [10]. Sequence analysis revealed 81% N protein identity between these isolates, while they could additionally be distinguished based on their biology (host range) and serology [10]. Such observations helped to establish the 90% threshold for species demarcation. In 2004 [11], the *Gn* and *Gc* genes of BR-03 and SA-05 were sequenced and revealed 92% aa identity. However, when looking in further detail, the phylogenetic distance between BR-03 and SA-05 based on the glycoprotein sequences was similar to that observed between tomato spotted wilt virus (TSWV) isolates, which was unexpected and questioned the N protein-based species demarcation [11]. The real meaning of this higher identity between the glycoproteins of TCSV and GRSV had been overlooked until a recent report on a proposed first interspecific reassortant tospovirus collected in the United States (U.S.) [12]. This isolate contained the S and L segments from GRSV and was proposed to contain the M segment from TCSV. Since all viruses/isolates presented this genomic configuration and their parental genotypes have not been found, the authors suggested that GRSV/TCSV isolates were introduced in the U.S. as reassortants. To support the assumption that the M segment of these reassortants originated from TCSV, the authors used the glycoproteins of BR-03 and SA-05 for comparison, considering them as parental genotypes [12]. 

From all these results abovementioned, we started wondering whether all GRSV and TCSV isolates sequenced so far have been sharing a highly conserved M segment and, more importantly, whether the BR-03 and SA-05 isolates could be considered as parental genotypes of TCSV and GRSV, respectively. To increase the reliability of our analyses, the complete genome of the original BR-03 and SA-05 isolates were high-throughput sequenced in this work. After several analyses, our results suggested that TCSV may have incorporated the M segment of GRSV and that the parental genotype of TCSV was eliminated or remains to be identified.

## 2. Materials and Methods

### 2.1. Sample Preparation and Sequencing

*Nicotiana benthamiana* leaf material infected with isolates BR-03 (TCSV) or SA-05 (GRSV) were kept frozen (−80 °C) as a virus inoculum stock since the early 1990s at the Wageningen University, Netherlands. These frozen leaves were ground and used for mechanical inoculation of wild type *N. benthamiana* plants as previously described [13]. Inoculated plants were kept in greenhouse until the onset of symptoms (two weeks). Total RNA of symptomatic (systemic) leaves was extracted with RNeasy Plant Mini Kit (Qiagen, Hilden, Germany) following the manufacturer’s instructions. High-throughput sequencing was performed on a HiSeq™ 2000 platform (2 × 100 bp read length) at Macrogen (Seoul, South Korea).

### 2.2. De Novo Assembly of Virus Genomes

Reads generated by the Illumina platform were trimmed and de novo assembled using the software CLC Genome Workbench 6.5.2 (CLC bio, Aarhus, Denmark). Contigs corresponding to virus sequences were identified using Blastx [14] against a Refseq virus database. Alignments with other tospoviruses were then performed using the program Geneious R8 (Biomatters, Auckland, New Zealand) to evaluate if the assembled contigs corresponded to the complete genomes of BR-03 and SA-05.

### 2.3. Phylogenetic Analyses

A preliminary neighbor-joining tree was constructed using fragments of *NSm* genes from TCSV, GRSV, and TSWV isolates obtained from the NCBI portal using 1000 bootstrap replicates. TSWV sequences were included in this analysis for comparison. Maximum likelihood (ML) trees were constructed using the amino acid sequences coded by the S, M, and L segments of TCSV, GRSV, and TSWV available at the NCBI portal. For the S and M segments that code for more than one protein, the amino acid sequences were concatenated. Only tospovirus isolates with all open reading frames (ORFs) sequenced were included in this analysis. As done previously, TSWV isolates were included for comparison since they display an intraspecific genetic distance between each other. Multiple alignments were done using the program MUSCLE [15] and the phylogenetic trees were built using the software PhyML v3.2 [16] (bootstrap = 1000 replicates), both implemented in Geneious R8. The program ProtTest v.3.4.2 [17] was used to estimate the best substitution model for all ML phylogenetic trees, which were then visualized and edited using the program FigTree v1.3.1. Heat maps for pairwise amino acid identities were built using the program SDT [18].

### 2.4. Evaluation of Synonymous-Site Variability and Nucleotide Diversity

To evaluate the accumulation of silent mutations along the S, M, and L segments of TCSV and GRSV, the synonymous-site variability was estimated with the program SynPlot2 [19] using the concatenated ORFs sequences. The program DNASP [20] was used to estimate nucleotide diversity parameters between TCSV and GRSV. Due to the low number of available sequences from the L segment of TCSV and GRSV, only fragments of *N* and *NSm* genes were included in this analysis.

### 2.5. TMRCA Calculation

The time to most recent common ancestor (TMRCA) between TCSV and GRSV was estimated by Markov chain Monte Carlo (MCMC) Bayesian analysis for each segment using BEAST v2.5.0 [21]. Due to the low number of TCSV and GRSV sequences, TSWV was also included in this analysis to estimate the substitution rates using only coding genomic regions. The temporal structure of the sequences was investigated with TempEst v1.6.0 [22] prior to the MCMC runs. Using only *NSm* genes for the M segment resulted in a better association between genetic distance and sampling dates, possibly due to the high number of these sequences on public databases. Trees were inferred under an uncorrelated lognormal relaxed molecular clock and a Bayesian Skyline tree prior [23,24]. The package bModelTest [25] was used as a site model to average over substitution models during the MCMC runs. Convergence of the parameters was determined by its effective sample size (ESS) with the program Tracer v1.6.0 and 10% of the samples of each run was discarded as burn-in.

## 3. Results

**Figure 1:**
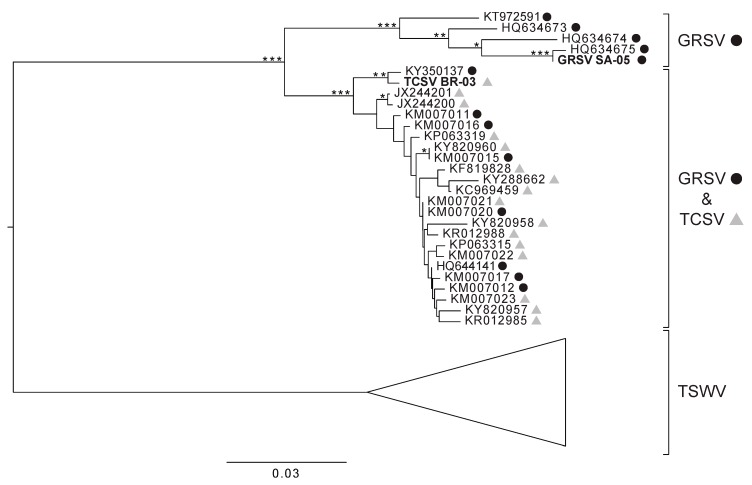
Phylogenetic tree based on *NSm* gene fragments from groundnut ringspot virus (GRSV), tomato chlorotic spot virus (TCSV), and tomato spotted wilt virus (TSWV) isolates. TCSV sequences are represented by grey triangles next to their accession numbers, while GRSV sequences are represented by black circles. Nodes with bootstrap values above 50%, 70%, and 90% are indicated by one, two, and three asterisks, respectively.

The majority of sequences available on public databases that match the M segment of TCSV and GRSV isolates correspond to fragments of *NSm* genes. Thus, these sequences were used to evaluate whether TCSV and GRSV isolates indeed share a highly identical M segment. Regarding genetic distance, *NSm* genes of TSWV isolates (most abundant on public databases) were included for comparison. Interestingly, the trees generated on the *NSm* genes showed that the interspecific diversity between TCSV and GRSV isolates is comparable to the intraspecific diversity between TSWV isolates (Figure 1). Additionally, the GRSV isolates segregated in two groups, one containing only GRSV isolates and another in which GRSV isolates intercalated with TCSV isolates. 

**Figure 2:**
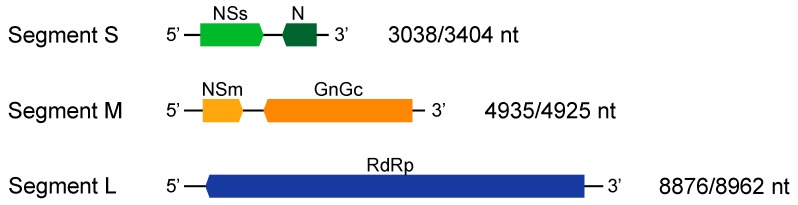
Genomic organization of TCSV and GRSV. The numbers on the right indicate the length of each segment of isolates SA-05 (GRSV) and BR-03 (TCSV), respectively.

By this work, there have been only partial sequences of the isolates BR-03 (TCSV) and SA-05 (GRSV) available on public databases. To circumvent this problem and further substantiate our findings, we have sequenced the complete genome of these isolates from infected *N. benthamiana* leaf material kept frozen at −80 °C. After de novo assembly, both genomes presented the standard tripartite single-stranded RNA pattern as seen in Figure 2. The S, M, and L RNA segments of each isolate were deposited in GenBank, respectively; (i) MH742961, MH742960, and MH742959 for BR-03 (TCSV) and (ii) MH742958, MH742957, and MH742956 for SA-05 (GRSV).

**Figure 3:**
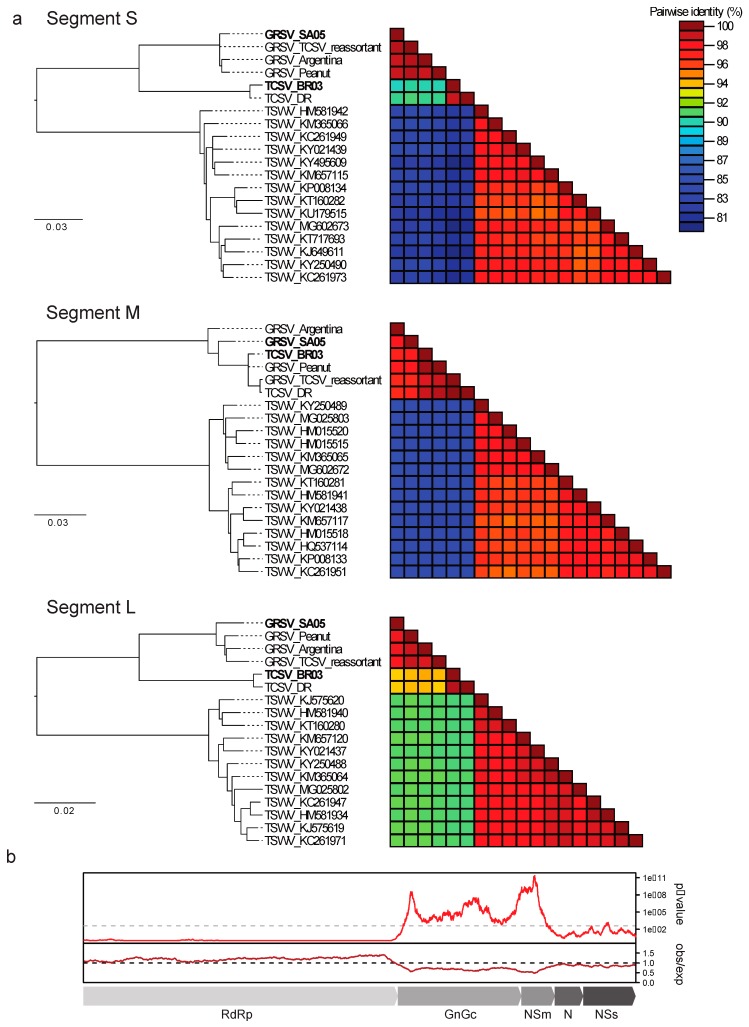
Phylogenetic trees based on concatenated protein sequences encoded in the S, M, and L RNAs of TCSV, GRSV, and TSWV isolates, and the observed/expected synonymous mutations in the coding regions of TCSV and GRSV isolates. (**a**) Maximum likelihood trees and protein identity plots of the S, M, and L segments of TCSV, GRSV, and TSWV and (**b**) suppression of synonymous mutation variability in the concatenated ORFs of TCSV and GRSV using a sliding window of 250 codons.

With the availability of BR-03 and SA-05 genomes, phylogenetic trees were built with protein sequences derived from the three RNA segments. In the trees based on protein sequences from the S and L segments, TCSV and GRSV isolates clustered separately, forming two groups (Figure 3a). In contrast, in the phylogenetic tree based on proteins from M segments, TCSV isolates intercalated in a single group together with a previously assumed reassortant [12] and a GRSV isolate recently identified in peanut in Brazil (Figure 3a). The highest identity between a TCSV isolate and a GRSV isolate was 89.2 % for the S segment (N and NSs proteins), 93.8% for the L segment (RdRp protein), and 99.8% for the M segment (Gn, Gc, and NSm proteins). 

To investigate whether the M segments from TCSV and GRSV isolates have accumulated less silent mutations in comparison with the S and L segments, and are, therefore, more closely related, we evaluated the suppression of synonymous mutations of the coding regions in the whole genome of both TCSV and GRSV isolates. Synonymous site variability of *L* (L segment) and of *NSs* and *N* (S segment) was within the expected value with an observed/expected ratio ~ 1 or > 1. Differently, the *Gn*, *Gc*, and *NSm* genes (M segment) showed an observed/expected ratio < 1 with high significance as seen in the *p*-value graphic (Figure 3b). To verify whether TCSV isolates accumulated less diversity overtime than GRSV isolates, nucleotide diversity parameters were estimated using the program DnaSP [20]. These analyses revealed that the M segments of TCSV isolates presented lower nucleotide diversity than those from GRSV isolates (Table 1).

To test whether the M segment from TCSV and GRSV are indeed more closely related than the S and L segments, the TMRCA was estimated by Bayesian phylogenetic analysis. The mean substitution rates were similar, with 2.4392E-4, 2.9163E-4, and 2.1783E-4 substitutions/site/year for the S, M (*NSm*), and L segments, respectively. Additionally, they presented narrow 95% high posterior density (95% HPD) intervals: 7.8493E-5 to 4.0956E-4, 1.5095E-4 to 4.4141E-4, and 1.1819E-4 to 3.2171E-4 for the S, M (*NSm*), and L segments, respectively. The mean TMRCA estimated between TCSV and GRSV was 548.52 years (95% HPD interval: 212.54 to 1065.5) for the S segment and 571.68 years (95% HPD interval: 305.03 to 933.48) for the L segment. Interestingly, but somewhat expected, the mean TMRCA of the M segment was much more recent, being 101.95 years (95% HPD interval: 57.71 to 154.47). The fact that the 95% HPD interval of the TMRCA between GRSV and TCSV for the M segment do not overlap with those from the S and L segments states that the TMRCA of the M segment is more recent. Focusing on the cluster containing intercalated GRSV and TCSV isolates (M segment), the first reported TCSV reassortment event may have happened about 38.01 years ago (95% HPD interval 29.71 to 48.43) as seen in Supplementary Materials Figure S1. The mean TMRCA of TSWV was 136.99 (95% HPD interval: 56.82 to 258.87), 78.07 (95% HPD interval: 43.53 to 124), and 121.15 (95% HPD interval: 69 to 193.24) years for the S, M, and L segments, respectively. Note that although the mean TMRCA for the M segment of TSWV is also more recent than for S and L segments, their 95% HPD interval overlap. 

## 4. Discussion

The evolutionary history of TCSV and GRSV isolates has been inferred in this work. For our analyses, we have sequenced the complete genomes of the first isolates (BR-03 and SA-05) of these viruses as they were in the 1990s. The focus was to understand why they share a highly conserved M segment, while their S and L genomic segments are more genetically distant. Previous works have observed this trait between these two tospoviruses [12,26]. While these previous studies drew their conclusions from protein identity analysis, we examined the diversity accumulation in the coding regions of the three viral genomic segments and provided a refined phylogenetic analysis comparing GRSV, TCSV, and TSWV isolates.

Our analyses revealed that the TCSV and GRSV M segments exhibit the lowest diversity accumulation in comparison with the S and L segments. There are two possibilities that could explain this variation. Either the coding sequences of the M segment are highly functional at the RNA level, constraining the introduction of mutations, or a reassortment event took place. The first possibility seems very unlikely due to the fact that the substitution rate estimated is similar for the three RNA segments. Based on the nucleotide diversity analysis, the M segment of TCSV isolates presented less diversity accumulation than GRSV isolates, suggesting that the TCSV isolates are reassorted tospoviruses. In previous works, GRSV isolates found in the U.S were reported as reassortants, containing the M segment of TCSV [12,27]. To come up with their conclusions, the authors considered both BR-03 and SA-05 isolates as parental genotypes. Our analyses, however, suggest that the parental genotype of TCSV still remains to be identified or it was eliminated since the M segment of BR-03 is not genetically distant enough to be regarded as such. 

Reassortment events appear to be frequent between TCSV and GRSV, given that TCSV sequences are scattered throughout the TCSV and GRSV cluster for *NSm* gene/M segment trees. The reason why this reassorted M segment was fixed in all known TCSV isolates remains to be investigated. In any case, it may have increased the virus adaptation to both plant and invertebrate hosts since the parental genotypes of TCSV have not been reported so far. It is worthy to notice that in recent years, TCSV have significantly increased its spread to other regions of the Americas [27,28,29,30,31,32] as a possible biological advantage of these recurrent interspecific reassortment events.

## Figures and Tables

**Table 1:** Nucleotide diversity analysis of TCSV and GRSV.

Protein	Virus	Number of Isolates	Number of Segregating Sites (S)	Average Number of Differences (K)	Nucleotide Diversity (π)	Nucleotide Diversity with Jukes Cantor Correction (π JC)
N (212 nt)	TCSV	28	43	5.26190	0.02482	0.02554
	GRSV	25	57	9.64333	0.04549	0.04742
	TCSV and GRSV	53	96	22.98621	0.10843	0.12246
NSm (381 nt)	TCSV	16	27	4.28333	0.01124	0.01135
	GRSV	13	48	16.69231	0.04381	0.04581
	TCSV and GRSV	29	66	11.20936	0.02942	0.03066

## Supplementary Materials

The following is available online at http://www.mdpi.com/1999-4915/11/2/187/s1, Figure S1: Bayesian phylogenetic analysis of the S, M (*NSm*), and L segments of TCSV, GRSV, and TSWV.
