# Supplementary material for: Tomato Chlorotic Spot Virus (TCSV) Putatively Incorporated a Genomic Segment of Groundnut Ringspot Virus (GRSV) Upon a Reassortment Event
Source: Viruses. 2019 Feb 22;11(2):187. doi: 10.3390/v11020187 (PMC6410062; doi:10.3390/v11020187)
Supplement: Supplementary file 1 [file viruses-11-00187-s001.pdf]

Maximum clade credibility tree of the S segment from TCSV, GRSV and TSWV

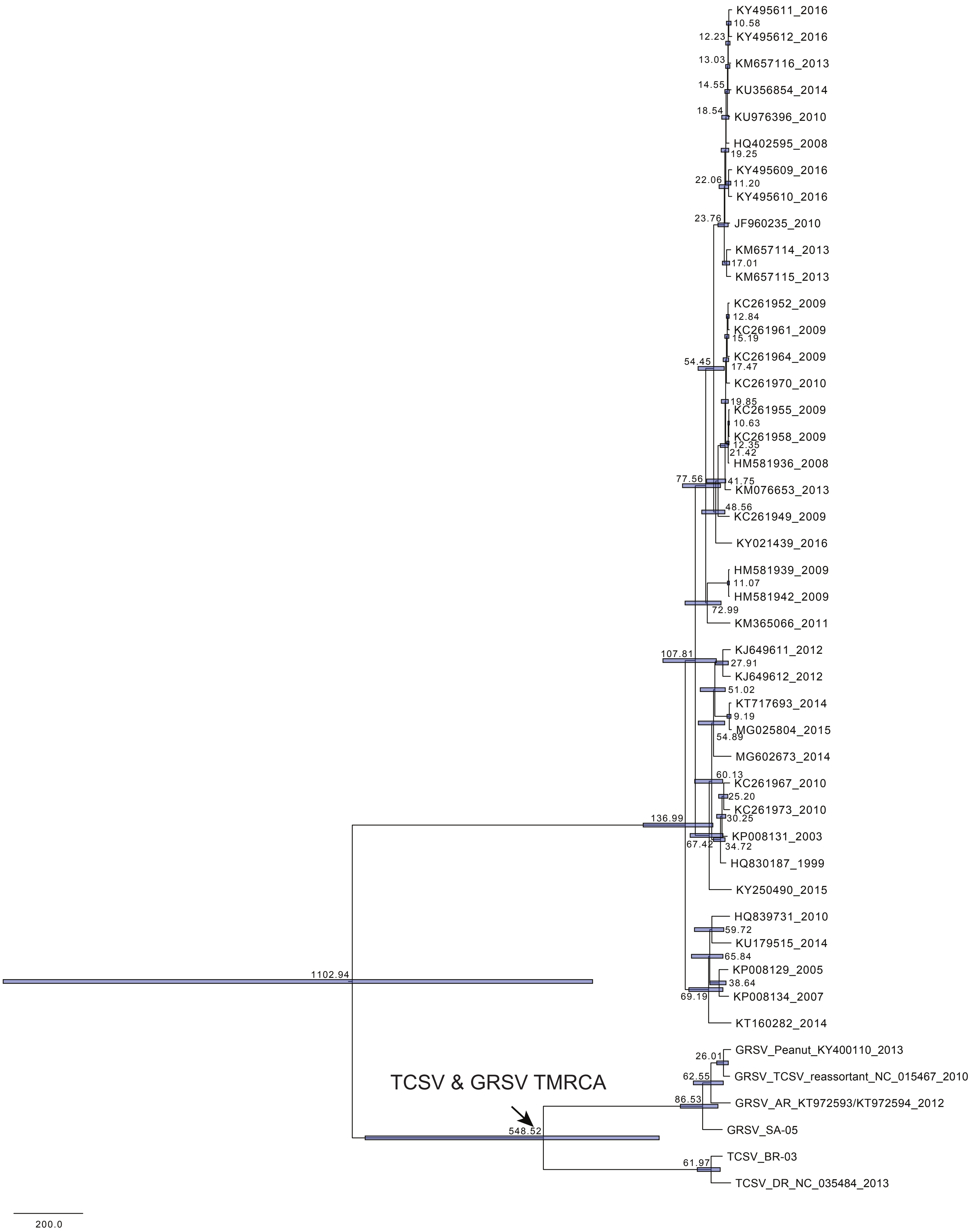

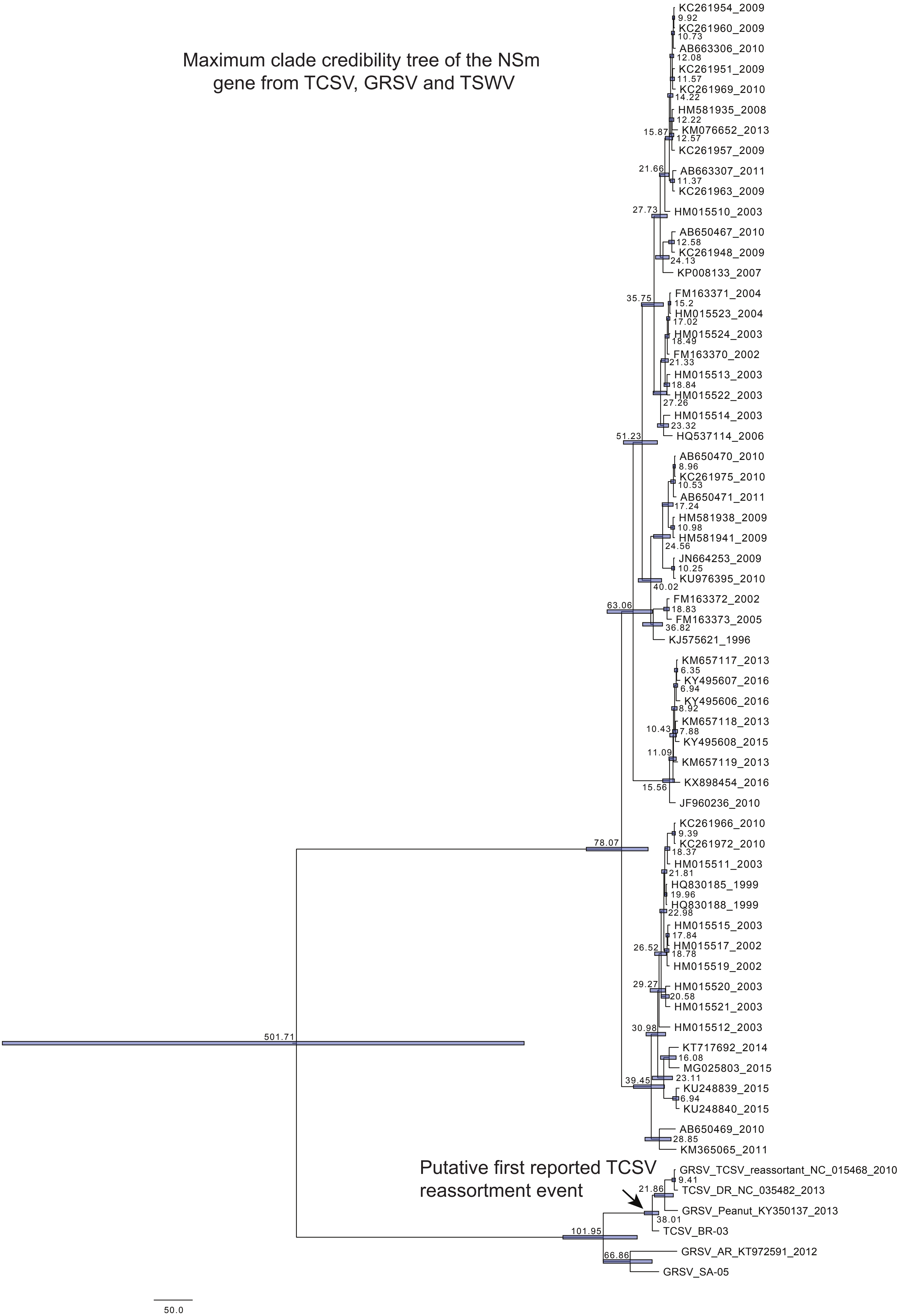

Maximum clade credibility tree of the NSm gene from TCSV, GRSV and TSWV
KC261954_2009
KC261960_2009
AB663306_2010
KC261951_2009
KC261969_2010
HM581935_2008
KM076652_2013
KC261957_2009
AB663307_2011
KC261963_2009
HM015510_2003
AB650467_2010
KC261948_2009
KP008133_2007
FM163371_2004
HM015523_2004
HM015524_2003
FM163370_2002
HM015513_2003
HM015522_2003
HM015514_2003
HQ537114_2006
AB650470_2010
KC261975_2010
AB650471_2011
HM581938_2009
HM581941_2009
JN664253_2009
KU976395_2010
FM163372_2002
FM163373_2005
KJ575621_1996
KM657117_2013
KY495607_2016
KY495606_2016
KM657118_2013
KY495608_2015
KM657119_2013
KX898454_2016
JF960236_2010
KC261966_2010
KC261972_2010
HM015511_2003
HQ830185_1999
HQ830188_1999
HM015515_2003
HM015517_2002
HM015519_2002
HM015520_2003
HM015521_2003
HM015512_2003
KT717692_2014
MG025803_2015
KU248839_2015
KU248840_2015
AB650469_2010
KM365065_2011
Putative first reported TCSV reassortment event
GRSV_TCSV_reassortant_NC_015468_2010
TCSV_DR_NC_035482_2013
GRSV_Peanut_KY350137_2013
TCSV_BR-03
GRSV_AR_KT972591_2012
GRSV_SA-05
501.71
78.07
101.95
66.86
63.06
51.23
50.0

Maximum clade credibility tree of the L segment from TCSV, GRSV and TSWV

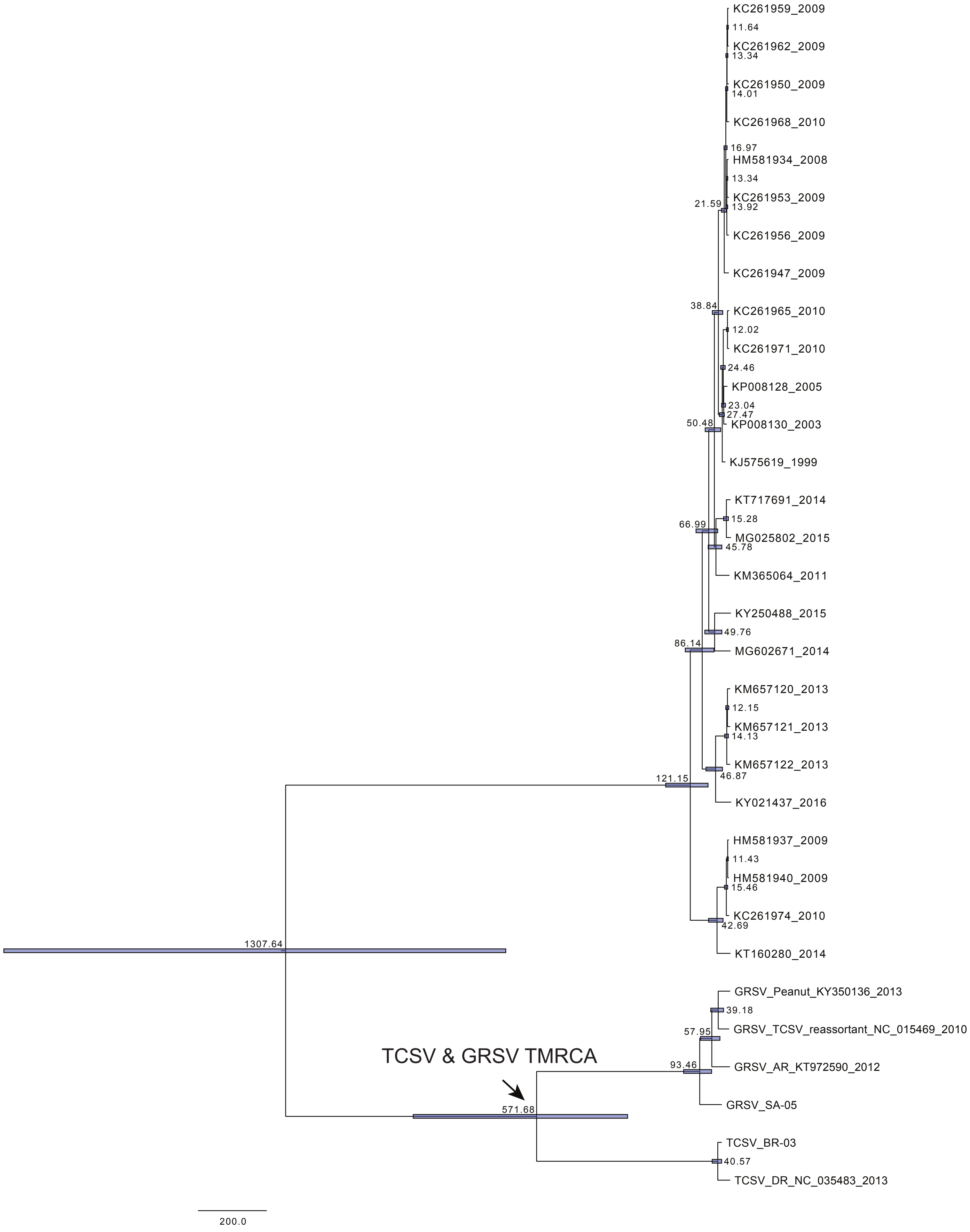

Figure S1. Bayesian phylogenetic analysis of the S, M (NSm) and L segments of TCSV, GRSV and TSWV. Maximum clade credibility trees of the S, M (NSm) and L RNAs of TCSV, GRSV and TSWV. Node heights represent the mean heights of each MCMC run. Blue bars represent the 95% HPD interval of the tree heights. The TMRCA between TCSV and GRSV is shown on the S and L segment trees, and the putative first reassortment event between TCSV and GRSV is shown on the NSm tree.
